# Genetic risk factors for the posterior cortical atrophy variant of Alzheimer's disease

**DOI:** 10.1016/j.jalz.2016.01.010

**Published:** 2016-08

**Authors:** Jonathan M. Schott, Sebastian J. Crutch, Minerva M. Carrasquillo, James Uphill, Tim J. Shakespeare, Natalie S. Ryan, Keir X. Yong, Manja Lehmann, Nilufer Ertekin-Taner, Neill R. Graff-Radford, Bradley F. Boeve, Melissa E. Murray, Qurat ul Ain Khan, Ronald C. Petersen, Dennis W. Dickson, David S. Knopman, Gil D. Rabinovici, Bruce L. Miller, Aida Suárez González, Eulogio Gil-Néciga, Julie S. Snowden, Jenny Harris, Stuart M. Pickering-Brown, Eva Louwersheimer, Wiesje M. van der Flier, Philip Scheltens, Yolande A. Pijnenburg, Douglas Galasko, Marie Sarazin, Bruno Dubois, Eloi Magnin, Daniela Galimberti, Elio Scarpini, Stefano F. Cappa, John R. Hodges, Glenda M. Halliday, Lauren Bartley, Maria C. Carrillo, Jose T. Bras, John Hardy, Martin N. Rossor, John Collinge, Nick C. Fox, Simon Mead

**Affiliations:** a Department of Neurodegenerative Disease, Dementia Research Centre, UCL Institute of Neurology, London, UK; b Department of Neuroscience, Mayo Clinic, Jacksonville, FL, USA; c Department of Neurodegenerative Disease, MRC Prion Unit, UCL Institute of Neurology, London, UK; d Department of Neurology, Mayo Clinic, Jacksonville, FL, USA; e Department of Neurology, Mayo Clinic, Rochester, MN, USA; f UCSF, San Francisco, CA, USA; g Memory Disorders Unit, Department of Neurology, University Hospital Virgen del Rocio, Seville, Spain; h Institute of Brain, Behaviour and Mental Health, University of Manchester, UK; i Alzheimer Center, Department of Neurology, VU University Medical Center, Neuroscience Campus, Amsterdam, Netherlands; j Department of Epidemiology & Biostatistics, VU University Medical Center, Amsterdam, The Netherlands; k UC San Diego/VA San Diego Healthcare System, San Diego, CA, USA; l INSERM U610, Hôpital de la Salpêtrière, Paris, France; m Centre des Maladies Cognitives et Comportementales, IM2A, ICM, Paris 6 University, France; n Regional Memory Centre (CMRR), CHU Besançon, Besançon, France; o University of Milan, Fondazione Cà Granda, IRCCS Ospedale Policlinico, Italy; p Vita-Salute San Raffaele University, Milan, Italy; q University of New South Wales, Sydney, Australia; r Alzheimer's Association, Chicago, IL, USA; s Department of Molecular Neurosciences, UCL Institute of Neurology, London, UK

**Keywords:** Posterior cortical atrophy, Alzheimer's disease, Genetics, GWAS, Selective vulnerability, *APOE*

## Abstract

**Introduction:**

The genetics underlying posterior cortical atrophy (PCA), typically a rare variant of Alzheimer's disease (AD), remain uncertain.

**Methods:**

We genotyped 302 PCA patients from 11 centers, calculated risk at 24 loci for AD/DLB and performed an exploratory genome-wide association study.

**Results:**

We confirm that variation in/near *APOE/TOMM40* (*P* = 6 × 10^−14^) alters PCA risk, but with smaller effect than for typical AD (PCA: odds ratio [OR] = 2.03, typical AD: OR = 2.83, *P* = .0007). We found evidence for risk in/near *CR1* (*P* = 7 × 10^−4^), *ABCA7* (*P* = .02) and *BIN1* (*P* = .04). ORs at variants near *INPP5D* and *NME8* did not overlap between PCA and typical AD. Exploratory genome-wide association studies confirmed *APOE* and identified three novel loci: rs76854344 near *CNTNAP5* (*P* = 8 × 10^−10^ OR = 1.9 [1.5–2.3]); rs72907046 near *FAM46A* (*P* = 1 × 10^−9^ OR = 3.2 [2.1–4.9]); and rs2525776 near *SEMA3C* (*P* = 1 × 10^−8^, OR = 3.3 [2.1–5.1]).

**Discussion:**

We provide evidence for genetic risk factors specifically related to PCA. We identify three candidate loci that, if replicated, may provide insights into selective vulnerability and phenotypic diversity in AD.

## 1 Introduction

Posterior cortical atrophy (PCA) is a rare neurodegenerative syndrome, typically a variant of Alzheimer's disease (AD), although occasionally due to other pathologies including dementia with Lewy bodies, corticobasal degeneration, and prion disease [1]. Patients with PCA present with combinations of cognitive problems attributable to posterior cortical dysfunction and in particular difficulties with higher level visual processing including simultanagnosia, optic apraxia, optic ataxia, and visual disorientation; other features may include dyslexia, dyscalculia, dysgraphia, and limb dyspraxia. In contrast with typical, amnestic AD, memory is relatively spared until the disease becomes advanced. MR brain imaging in PCA typically shows parieto-occipital lobe atrophy with relative preservation of medial temporal lobe structures [2]; fluoro-deoxyglucose positron emission tomography (PET) shows prominent posterior cortical hypometabolism [3]; and in a single case study using the AV1451 tau PET tracer, posterior cortical tau deposition [4]. By contrast, PET imaging using amyloid-binding ligands typically shows global amyloid deposition [3]. Aside from the imaging and cognitive differences, patients with PCA are typically younger than those with typical amnestic late-onset AD, usually with disease onset in the sixth or seventh decade [1]. PCA is almost invariably a sporadic disorder, and the risk factors for developing the syndrome are unknown. Understanding the genetic architecture of the PCA variant of AD may provide insights both into factors predisposing to young onset AD, as well as mechanisms underlying regional vulnerability in AD.

To date, only a few, single-center studies have addressed genetic risk for PCA [5], [6], [7], [8], [9], [10], and due to the rarity of the syndrome all have been relatively small, the largest being a maximum of 81 cases [9]. Some, but not all, of these studies have suggested that despite their early-disease onset, patients with PCA may be less likely than expected to have an *APOE* ε4 allele, the commonest risk factor for late-onset AD. Other studies have suggested that there may be differences in some of the more recently identified genetic risks for AD in patients with PCA [9]. Recognizing the rarity of this AD variant, we formed an international consortium comprising eleven centers, using clinical diagnostic criteria to define cases of PCA, with the principal aim of determining whether *APOE* ε4 and genetic risks from recent genome-wide association studies (GWAS) of AD and dementia with Lewy bodies (DLB, see below) are risk factors for the PCA variant of AD. In a second, exploratory analysis, we performed a pilot GWAS analysis to identify novel putative genetic risk factors for PCA.

## 2 Methods

### 2.1 PCA patients and controls

After an inaugural multidisciplinary meeting of PCA researchers [11], latterly formalized as the Alzheimer's Association's International Society to Advance Alzheimer's Research and Treatment (ISTAART) Professional Interest Area in Atypical AD and Associated Syndromes, an international collaborative group was established to assess genetic risk factors for PCA. Researchers identified individuals with PCA, in whom a deoxyribo nucleic acid (DNA) sample was available. Patients who the referring physician had diagnosed with AD, had multidomain cognitive impairment fulfilling criteria for AD dementia, and had one or both of two published criteria for PCA, as proposed by Tang-Wai [5] and Mendez [12] (Table 1) were included. Additional data collected included gender, age at disease onset, age at death (where applicable), and whether there was molecular (cerebrospinal fluid or amyloid PET using locally defined ranges) evidence or pathologic confirmation of underlying AD pathology. Each site had appropriate local ethical approvals in place, and all participants gave informed written consent. Controls were from UK, USA, and Germany (see below).

### 2.2 Genetic and statistical analyses

DNA samples were analyzed at the MRC Prion Unit, Department of Neurodegenerative Disease, Institute of Neurology, UCL. PCA samples were genotyped on Illumina 660 arrays (n = 54, UCL cohort only) and OmniExpress arrays (n = 239, all cohorts); in total, 293 passed sample quality control, implemented using PLINK. Controls were genotyped on Illumina 550 (n = 809, KORA F4 German, PMID: 16032514), OmniExpress (n = 1185, Geisinger US http://www.geisinger.org), Illumina 2.5 M (n = 1882, KORA F3 German), Illumina 5M (n = 1651, Framingham US, see acknowledgments), and Illumina 1.2 M (n = 5020, WTCCC2 UK, see acknowledgments) arrays. Physical locations refer to the Feb 2009 (GRCh37/hg19) assembly. We excluded SNPs with a minor allele frequency (MAF) < 1% (n = 70,042 from OmniExpress case arrays); genotyping rate <99% (n = 85,086 from OmniExpress case arrays); or Hardy-Weinberg Equilibrium (HWE) exact test *P* < 10^−3^ in controls. Cases with call rate <98% (n = 6) as well as ethnic outliers (n = 2) were excluded after visualization of multidimensional scaling plots. Related and duplicate cases were removed by IBS/IBD calculation (n = 1) and re-examination of patient data, as they became available post genotyping (n = 2). A Pi-Hat (proportion identity by descent) threshold of >0.1875 was used, which should exclude first and second degree relatives. Two duplicates removed due to later availability of patient data were also included within the six cases removed for low call rate thus bringing the total number of cases removed to nine. Owing to the multitude of different genotyping platforms, control comparisons were carried out sequentially and exclusions removed at each stage. A total of 840 KORAF4 German controls were originally genotyped; 17 with call rate <98%, six with Pi-Hat > 0.1875 and eight MDS outliers were removed. A total of 1950 KORAF3 German controls were originally downloaded; three with call rate <98%, 57 with Pi-Hat > 0.1875, and eight MDS outliers were removed. A total of 1264 Geisinger US controls were originally downloaded; two with call rate <98%, 69 with Pi-Hat >0.1875, and eight MDS outliers were removed. 2467 FHS US controls were originally downloaded; 16 with call rate <98%, 793 with Pi-Hat >0.1875 and seven MDS outliers were removed. 5050 WTCCC2 UK controls were generated from available raw IDAT files; 26 with call rate <98%, four with Pi-Hat > 0.1875, and zero MDS outliers were removed (see Supplementary Table 1). All remaining cases and controls were finally visualized on an MDS plot (see Supplementary Fig. 1), outlier detection was performed using PLINK v1.07, and no further outliers were detected. IBS/IBD estimation of the final cases and controls also lead to no further exclusions based on relatedness. Shapeitv2 was used, in conjunction with the 1000 Genomes Phase 1 Integrated variant set (b37 March 2012 release), to align all data relative to the positive strand [13]. To avoid potential downstream cross platform confusion, however, we removed any A/T or G/C transversions to phase each chromosome from each platform separately before imputation using Impute2 (v2.3.0). GTOOL (v0.6.6) was used to extract and collate samples into their respective cohorts for association testing [14]. Association testing was performed using SNPtest_v2.5-beta4 employing the frequentist (additive model) score method which involves weighting by the likelihood of each imputed genotype [15]. Four population covariates derived from IBS/IBD analysis in PLINK were used in the association analysis [16], [17]. The case-control association test statistic inflation factor was 1.06 (Supplementary Fig. 2). Any association statistics mentioned in the results section are shown with standard genomic control (*P*_*GC*_) corrected and uncorrected *P* values. Post association-testing QC excluded markers with a MAF <1% and departure from HWE, in both combined and any single control cohort *P* < 10^−4^. We also excluded markers with a SNPtestv2 derived “info metric” and “add info metric” below 0.9. The final autosomal analysis thus included 5.9 M markers.

We assessed whether the genetic risks for typical AD and PCA were different by comparing odds ratios (OR) between our study and those published in typical AD [18]. We employed a Wald-type test, first calculating the standard error of the difference in log (OR) from the reported CIs. We then divided the empirical difference in log (OR) by its standard error, and thus derived a z-score and *P* value, relying on the approximate normality of log (OR) estimates from large samples. In the main analysis of candidate SNPs, we used a Bonferroni corrected association threshold of *P* < .002 based on the testing of a lead SNP from 24 independent loci derived from studies of AD [18] and DLB [19].

Although the typical samples sizes that are required to discover novel genome-wide significant risk factors in complex disorders are in the thousands, there are some precedents of strong genetic effects detected with small but phenotypically homogenous samples, including variants at *APOE* in AD [20], *PRNP* in prion disease [21], and complement factor H in age related macular degeneration [22]. We therefore performed an exploratory genome-wide association study using established methodologies that account for population structure and with imputation of SNPs not present on the genotyping arrays. As PCA is a clinical syndrome that may be due to pathologies other than AD, we also assessed the odds ratios at SNPs of interest in a subset of patients with biomarker/pathological evidence for AD.

## 3 Results

### 3.1 Patients and demographics

A total of 302 samples fulfilling entry criteria to the study were available from eleven centers: University College London (n = 94); Mayo Clinic, USA (n = 77); University of California San Francisco, USA (n = 25); University of Seville, Spain (n = 25); University of Manchester, UK (n = 20); VU University, Netherlands (n = 17); University of California San Diego, USA (n = 16); INSERM, France (n = 10); University of Milan, Italy (n = 6); CHU Besançon, France (n = 6); and University of New South Wales, Australia (n = 6). Demographics are shown in Table 2. All samples fulfilled Tang-Wai clinical criteria for PCA [5], and in the 225 samples for which data were available, 97.3% also fulfilled Mendez criteria [12]; 41% of the cohort was male. Mean (±SD) age at symptom onset was 58.9 ± 6.9 years, and 82.5% had young onset dementia, as defined by age at onset of <65 years. Thirty-four patients had died, with a mean age at death of 68.0 (±7.7) years. Molecular or pathologic evidence for underlying Alzheimer pathology was available for 82 (27%), of whom 52 had a CSF profile compatible with AD; 32 had a positive amyloid PET scan; and 15 had autopsy proven AD. None had evidence for pathology or biomarkers for non-AD pathology.

DNA from nine individuals failed array quality control, and statistical analyses were done on the remaining 293/302 samples (see Table 2). We also considered the associations in the sub-sample of 82 with biomarker/autopsy evidence for underlying AD pathology.

### 3.2 Comparisons at known genetic loci for AD and DLB

**Fig. 1:**
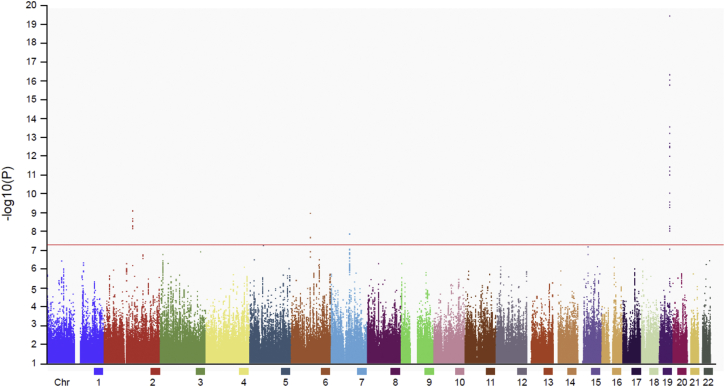
Manhattan plot of autosomes with the threshold for genome-wide significance (*P* < 5 × 10^−8^) indicated by the red line. Four loci achieved statistical significance at *APOE* (chromosome 19), *SEMA3C* (chromosome 7), *FAM46A* (chromosome 6), and *CNTNAP5* (chromosome 2).

**Fig. 2:**
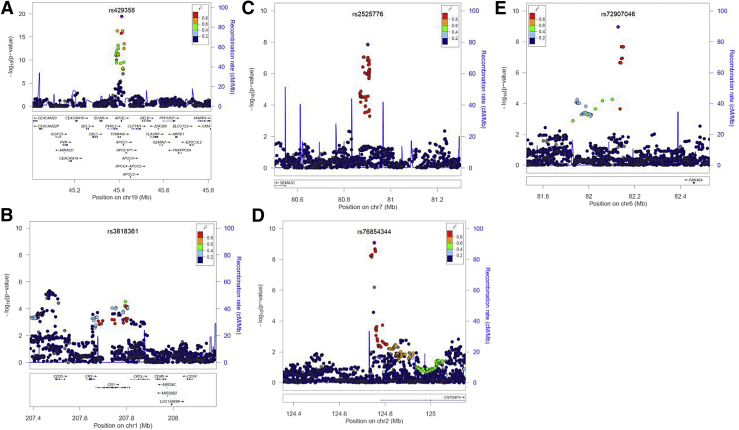
SNP annotation and proxy (SNAP) plots for five regions of interest showing significance in either known genetic loci for AD (A, B) or in the exploratory GWAS (A,C,D,E). These plots illustrate the statistical evidence of association at a locus together with information about nearby genes and linkage disequilibrium between the most strongly associated SNP and its neighbors on the chromosome.

Results of the genetic analysis of candidate risk factors for the whole PCA cohort are shown in Table 2. First, we considered 24 SNPs known to be genetic risk factors in AD and/or DLB. The best proxy genotyped for the *APOE* ε4 AD-risk allele, rs2075650, located on chromosome 19 in the *TOMM40* gene and 13kb upstream of *APOE*, was identified as a strong risk factor for PCA (OR 2.03, [95% CI = 1.68–2.46], *P* = 6 × 10^−14^, *P*_GC_ = 3 × 10^−13^; Fig. 1 and Fig. 2A). rs3818361 located on chromosome 1 in *CR1* was also significantly associated (ORs = 1.38 [1.14–1.67], *P* = 7 × 10^−4^, *P*_GC_ = 1 × 10^−3^; Fig. 2B). rs3764650 in *ABCA7* (OR = 1.39 [1.07–1.8], *P* = .02, *P*_GC_ = .02) and rs744373 upstream of *BIN1* (OR = 1.2 [1.01–1.43], *P* = .04, *P*_GC_ = .05) reached nominal significance but did not surpass our Bonferroni corrected threshold of *P* < .002. Other candidate SNPs showed no evidence of association.

In the subset of individuals with either biomarker (CSF or amyloid PET) or pathologic evidence for underlying AD (n = 82), rs2075650 (at the *APOE/TOMM40* locus, subsequently referred to as *APOE*) was again identified as a risk factor with a similar OR to the whole group (OR = 2.00 [1.39–2.89] *P* = 9 × 10^−5^, *P*_GC_ = 1 × 10^−4^). rs3818361 (*CR1*) and rs3764650 (*ABCA7*) both showed nominally significant differences compared with controls (*CR1* OR = 1.7 [1.20–2.41], *P* = .003, *P*_GC_ = .004; *ABCA7* OR = 1.83 [1.17–2.86], *P* = .009, *P*_GC_ = .01). There was no evidence for an effect of *BIN1* (OR = 1.08 [0.76–1.52], *P* = .65) in the biomarker cohort.

### 3.3 Comparing risk for PCA to typical AD

Comparing odds ratios and the nominal risk conferred in the whole PCA cohort against the most recently published mega-meta studies of typical AD [16], the effect size seen at the *APOE* locus was significantly less strong in PCA than for typical AD (PCA: OR 2.03, typical AD: OR 2.83, *P* = .0007, *P*_GC_ = .001, see methods). Although there was no evidence for risk in PCA vs controls, the risk effects at rs35349669 in *INPP5D* (*P* = .02, *P*_GC_ = .02) and rs2718058 upstream of *NME8* (*P* = .03, *P*_GC_ = .04) both were nominally different in PCA than typical AD.

### 3.4 Exploratory GWAS

As several different array platforms were used in the study, only 210,670 SNPs were genotyped across all samples. In this set, there was little evidence of inflation in the association test statistic (λ = 1.05). Only the proxy for the *APOE* ε4 AD-risk allele, rs2075650, achieved genome-wide significance. We went on to analyze 5.9-M SNPs in the imputed data set. Aside from chromosome 19 (*APOE* locus), three loci on chromosomes 7, 2, and 6 respectively were of interest (Fig. 1). rs2525776 on chromosome 7, upstream of *SEMA3C* (OR 3.3 [2.1–5.1], *P* = 1.4 × 10^−8^, *P*_GC_ = 4 × 10^−8^; Fig. 2C); rs76854344 on chromosome 2, upstream of *CNTNAP5*, (OR 1.9 [1.5–2.3], *P* = 8.0 × 10^−10^, *P*_GC_ = 2 × 10^−9^; Fig. 2D), and rs72907046 on chromosome 6, downstream of *FAM46A* (OR 3.2 [2.1–4.9], *P* = 1.1 × 10^−9^, *P*_GC_ = 3 × 10^−9^; Fig. 2E) were all associated with PCA. Restricting the analysis to the 82 individuals with biomarker/pathology evidence for underlying AD pathology, the corresponding odds ratios were similar: 3.8 [1.8–8.2], *P* = 2.8 × 10^−4^ for rs2525776; 1.8 [1.2–2.7], *P* = 2.1 × 10^−3^ for rs76854344; and 2.5 [1.0–6.1], *P* = 2.7 × 10^−2^ for rs72907046. None of these three loci showed any evidence of association with typical AD on the IGAP AD-risk GWAS meta-analysis. A full list of suggestive associations *P* < 10^−4^ based on the SNPs represented on case and control arrays (the intersection SNPs) is available in a Supplementary Table 2, and summary statistics from the entire data set is available at the NHGRI-EBI GWAS Catalog.

## 4 Discussion

We report findings from a consortium to study genetic risk factors in PCA, a rare predominantly early-onset cognitive disorder characterized by progressive and disproportionately posterior cortical dysfunction and atrophy, and usually associated with AD-type pathology. Our primary aim was to explore the relationship between PCA and a predetermined list of candidate SNPs derived from studies of typical AD and DLB. Our main findings are the identification of genetic risk for PCA at some of the known AD-risk loci, but not at the two DLB-risk loci that were tested (Table 3). We demonstrate PCA-risk association with variants in or near *APOE, CR1, ABCA7* and *BIN1* in the whole cohort, but only those at *APOE*, *CR1*, and *ABCA7* remain nominally significant in the small molecularly defined subgroup. We also show evidence for nonoverlapping CIs of genetic risk at *APOE, INPP5D*, and *NME8*. Although this is by far the largest study of PCA to date, our relatively small sample size remains underpowered to detect genome-wide significant associations at risk loci with small effect sizes, such as those shown to associate in AD-risk GWAS. However, our exploratory GWAS nominates the three novel loci, *SEMA3C*, *CNTNAP5*, and *FAM46A*, which achieved genome-wide significance, as potential genes of interest in PCA.

Although still small for genetic studies, this collection is the result of a global collaborative effort and is considerably larger than any previous studies of PCA reporting varying evidence that *APOE* is a risk factor [5], [6], [7], [8], [9], [10]. Although the vast majority of samples in previous studies overlap with the present study, individual studies comprise <∼25% of the current total. We found robust genetic association at *APOE* but with an odds ratio significantly smaller than those seen in typical AD. *APOE* is the best established and strongest risk factor for sporadic AD and has been associated not only with increased risk *per se*, but also with earlier disease onset [23], the rate of hippocampal atrophy [24] and more memory led disease. However, the situation is more complex in early-onset AD which is associated both with a greater proportion of nonamnestic presentations, and perhaps a relatively reduced proportion of *APOE* ε4 carriers [25]. Studies investigating *APOE* risk for PCA (or as it was sometimes previously defined, biparietal AD [6]), have shown mixed results, perhaps due to differences in case definition, and the limited sample size of each study. In this study of PCA, by far the largest yet published, we confirm that *APOE* is a risk factor for PCA, but that it is a weaker risk factor than for typical AD.

Addressing risk factors other than *APOE*, the largest single previous study—which included samples also used in this analysis—found nominally significant association with SNPs in or near *CLU*, *BIN1*, and *ABCA7* [9]. We found evidence of association in the same direction for the SNP at *ABCA7* both in the whole cohort and those with biomarker evidence for AD; for *BIN1* in the whole cohort alone; but could not confirm that SNPs near to *CLU* are risk factors. Aside from *APOE*, only variants in *CR1* surpassed our statistical threshold for multiple testing in the whole cohort; these variants also showed nominal significance in the biomarker positive subgroup. Estimates of effect size were greater in PCA than those in typical AD at each associated locus aside from *APOE*. Although these differences were only nominally statistically significant, the four risk loci we report are among the strongest known common genetic risk factors for typical AD (Table 3). *CR1* has multiple functions including the regulation of complement and phagocytosis of immune complexes and pathogens, which are increasingly though to be relevant to AD pathogenesis [26]. *ABCA7* may play a role in AD through regulation of phagocytosis or lipid metabolism. *BIN1* mechanisms in AD are unclear but may be involved in endocytosis and the recycling of endocytic vesicles [27].

Although they did not confer significant alteration of PCA risk, we found that odds ratio confidence intervals for SNPs at or near to *INPP5D* and *NME8* in PCA did not overlap those of typical AD and showed directionally opposite effects, *INPP5D* was identified as a risk factor for AD in a recent large meta-analysis and plays an important role in a number of inflammatory processes. There is little evidence for the function of *NME8* in the central nervous system, although a role in modification of oxidative stress has been proposed [28]. Although these findings were only nominally significant and need independent replication, they do raise the possibility that syndromic variants of AD may be differentially associated with alterations in certain risk genes, perhaps through altered responses to inflammation or stress.

The results of our exploratory genome-wide study implicate three potential strong risk loci, near to *CNTNAP5*, *FAM46A*, and upstream of *SEMA3C*. The regions of strong LD with these associations did not include directly genotyped SNPs across all platforms, and therefore false-positive associations related to differential accuracy of imputation between case and control arm of the study remain possible. With the caveat that these findings must therefore be considered preliminary and require follow-up replication in an independent sample and by direct genotyping, it is notable that all three genes have roles in processes potentially relevant to PCA. Contactin-associated protein-like 5 gene (*CNTNAP5*) belongs to a subgroup of the neurexin family of multidomain transmembrane proteins involved in cell adhesion and intercellular communication in the central nervous system and has been implicated as a risk factor for bipolar disorder and autism spectrum disorders [29]. Family with sequence similarity 46, member A1 (*FAM46A*), originally *C6orf37*, is preferentially expressed within the neural retina [30] and has been implicated in cell signaling pathways related to retinal neurodegeneration [31]. Class III semaphorins including Semaphorin 3C (*SEMA3C*) have been examined as potential modifying factors in neurodegeneration through interactions with plexins and neuropilins. *SEMA3C* has been identified as a chemotrophic molecule influencing attractive guidance for cortical axon development [32]; the expression of *SEMA3C* and its receptors have been shown to influence the maturation of the visual system [33]; and *SEMA3C* is also expressed in the hippocampus, where it has a role in influencing the afferent connections of the developing hippocampus and in particular the ingrowth of septo-hippocampal connections [34], the major cholinergic connections implicated in learning and memory [35]. Finally, *SEMA3C* expression has been shown to correlate with functional network connectivity within the brain [36]. Although at this stage speculative, it is possible therefore that perhaps subtle differences in cortical development might influence where pathology starts and/or how it spreads through the brain if a neurodegeneration process is initiated later in life. The fact that all three of these novel genetic risks showed nominal associations in the relatively small subset of individuals with biomarker evidence for AD and the absence of similar evidence of association in any of these genes with IGAP studies of typical AD suggest that if confirmed, these loci may be specific risks for PCA due to Alzheimer's disease.

The main limitation of our study is the necessarily modest sample size of this very rare disorder, noting that the case numbers presented here were only achievable through the establishment of an international consortium. We plan to continue to collect further samples to allow for replication in due course. Based on standard power calculations in case-control studies, even in the favorable situation of completely accurate imputation of the functional SNP we are only adequately powered to detect effect sizes of OR >1.5, for common candidate SNPs, and the only common genetic risk factor of this strength in typical AD is *APOE*. We made comparisons with studies of patients diagnosed with typical AD; however, these patients and/or studies are different in multiple ways including the genotyping platforms used, later age at clinical onset, potential pathologic heterogeneity, and most probably differences in geographical location, all of which could confound the comparison. Although PCA is underpinned by AD pathology in most cases, we only had evidence for underlying AD in a proportion (∼1/4), and we cannot confirm that the genetic risks we have determined are specific for the AD variant of PCA rather than the syndrome of PCA or for young onset AD. However, allowing for the fact that the confidence intervals are inevitably large, it is notable that the estimates for the odds ratios for *APOE*, *CR1*, and *ABCA7*, and the putative genes identified in our exploratory GWAS were similar or larger in the proportion with molecular evidence for AD, suggesting that the risk we identify are likely to be for the AD variant of PCA, rather than for the syndrome per se.

One of the major outstanding issues in neurodegenerative disease research is an explanation for the often very striking phenotypic heterogeneity underpinned by the same broad core pathology. Possibilities for phenotype modification include demographic and environmental factors, including age at onset, or perhaps more likely complex gene and/or environment interactions and/or factors related to the misfolded proteins and their propagation, tissue or network selectivity and toxicity. The results of this study suggest that subtle differences in established risk factors may be associated with some of this heterogeneity and provide testable suggestions for novel genes that may influence the development of the hippocampal and visual system, which may influence the development of the PCA phenotype relative to other syndromes. If confirmed in future studies, next generation sequencing may be useful in determining whether these findings might be underpinned by rare variants with large effect sizes. More broadly, genetic investigation of well-phenotyped AD variants may provide important insights into disease biology in typical AD.

**Research in context**

1. Systematic review: Reviewing the literature for publications investigating the genetics of posterior cortical atrophy (PCA), there is conflicting evidence for the role of *APOE* in the PCA variant of Alzheimer's disease AD), and limited evidence for the more recently identified genetic risks for AD.
2. Interpretation: Through the establishment of an international consortium to create the largest study exploring the genetics of PCA to date, we demonstrate that (1) *APOE* is a risk factor for PCA but confers a smaller risk than for typical AD; (2) some of the genetic risks for typical AD are also associated with PCA risk; and (3) nominate three novel risk loci for PCA.
3. Future directions: These data provide clear directions and testable hypotheses for future studies, including (1) the establishment of a replication cohort and (2) investigation of the identified genes as factors influencing selective vulnerability in AD.

## Figures and Tables

**Table 1:** Clinical criteria for PCA

Tang-Wai et al, 2004 [5]	Mendez et al, 2002 [11]
Core features Insidious onset and gradual progression Presentation of visual complaints in the absence of significant primary ocular disease Relative preservation of anterograde memory and insight early in the disorder Disabling visual impairment throughout the disorder Absence of stroke or tumor Absence of early parkinsonism and hallucinations *Any of the following findings* Simultanagnosia with or without optic ataxia or ocular apraxia Constructional dyspraxia Visual field defect Environmental disorientation Any of the elements of Gerstmann syndrome Supportive features Alexia Presenile onset Ideomotor or dressing apraxia Prosopagnosia Investigations Neuropsychological deficits referable to parietal and/or occipital regions Focal or asymmetric atrophy in parietal and/or occipital regions on structural imaging Focal or asymmetric hypoperfusion/hypometabolism in parietal and/or occipital regions on functional imaging.	Core diagnostic features (all must be present) Insidious onset and gradual progression Presentation with visual complaints with intact primary visual functions Evidence of predominant complex visual disorder on examination Elements of Balint's syndrome Visual agnosia Dressing apraxia Environmental disorientation Proportionally less impaired deficits in memory and verbal fluency Relatively preserved insight with or without depression Supportive diagnostic features Presenile onset Alexia Elements of Gerstmann's syndrome Ideomotor apraxia Physical examination within normal limits Investigations Neuropsychology: predominantly impaired perceptual deficits Brain imaging: predominantly occipitoparietal abnormality (especially on functional neuroimaging) with relative sparing of frontal and mesiotemporal regions.

**Table 2:** Clinical features and demographics

Total number of DNA samples received	302
Number (%) male	124 (41%)
Mean ± SD age at onset (y)	58.9 ± 6.9
Number (%) with young onset dementia (onset <65 y)	249 (83%)
Number with biomarker/path evidence for AD∗	82 (27%)
Number (%) with known age of death	34
Mean ± SD age at death (y)	67.9 ± 7.7
9 samples failed genetic QC	
Total number of DNA samples passing QC and entering analysis	293
Number (%) male	120 (41%)
Mean ± SD age at onset (y)	58.8 ± 6.9
Number (%) with young onset dementia (onset <65 y)	243 (83%)
Number with biomarker/path evidence for AD∗	77 (26%)
Number (%) with known age of death	33
Mean ± SD age at death (y)	67.8 ± 7.8

∗ No individual with biomarker/path evidence for a non-AD diagnosis was included.

**Table 3:** Results of the main analysis for candidate SNPs which were discovered in typical AD (ref [16], except ^∗^Seshadri et al. JAMA 2010:303; 1832–40, ^†^Hollingworth et al. Nat Genet 2011:43:429–35) or DLB [17]

Candidate SNP	Chr	Nearest gene	Typical AD/DLB OR	PCA vs control *P* value	PCA OR	PCA OR CI	*P* Value for comparison of OR (PCA vs typical AD)	Cases MAF	Control MAF
rs3818361	1	*CR1*	1.17	6.71E−04	1.38	(1.14–1.67)	.09	0.24	0.19
rs744373	2	*BIN1*	1.17	0.04	1.20	(1.01–1.43)	.77	0.32	0.28
rs35349669	2	*INPP5D*	1.08	0.21	0.89	(0.75–1.04)	.02	0.46	0.49
rs6825004	4	*SCARB2*	0.78	0.42	0.92	(0.77–1.1)	.38	0.29	0.31
rs7687945	4	*SNCA*	0.75	0.92	1.00	(0.85–1.18)	.08	0.49	0.49
rs190982	5	*MEF2C*	0.93	0.66	0.99	(0.83–1.18)	.50	0.40	0.40
rs10948363	6	*CD2AP*	1.10	0.67	0.97	(0.8–1.17)	.19	0.27	0.27
rs11767557	7	*EPHA1*	0.90	0.39	0.92	(0.74–1.13)	.73	0.19	0.20
rs2718058	7	*NME8*	0.93	0.17	1.12	(0.95–1.33)	.03	0.39	0.36
rs1476679	7	*ZCWPW1*	0.91	0.73	1.05	(0.88–1.25)	.12	0.31	0.30
rs11136000	8	*CLU*	0.87	0.27	0.91	(0.77–1.09)	.58	0.37	0.40
rs28834970	8	*PTK2B*	1.10	0.27	1.10	(0.93–1.3)	.98	0.37	0.35
rs10838725	11	*CELF1*	1.08	0.98	1.01	(0.84–1.2)	.45	0.32	0.31
rs670139^†^	11	*MS4A4E*	1.08	0.77	0.97	(0.82–1.14)	.19	0.40	0.41
rs983392	11	*MS4A6A*	0.90	0.71	1.04	(0.88–1.22)	.09	0.42	0.41
rs3851179	11	*PICALM*	0.87	0.39	0.94	(0.79–1.11)	.40	0.35	0.37
rs11218343	11	*SORL1*	0.77	0.57	0.81	(0.4–1.65)	.88	0.01	0.02
rs17125944	14	*FERMT2*	1.14	0.73	0.94	(0.7–1.27)	.21	0.08	0.09
rs10498633	14	*SLC24A4* *RIN3*	0.91	0.14	0.86	(0.7–1.06)	.62	0.20	0.23
rs3764650	19	*ABCA7*	1.20	0.02	1.39	(1.07–1.8)	.28	0.12	0.09
rs2075650	19	*APOE*	2.83	6.24E−14	2.03	(1.68–2.46)	.0007	0.25	0.14
rs3865444^†^	19	*CD33*	0.91	0.69	0.95	(0.8–1.14)	.61	0.30	0.31
rs597668^∗^	19	*EXOC3L2* *BLOC1S3* *MARK4*	1.18	0.59	1.04	(0.84–1.3)	.30	0.17	0.16
rs7274581	20	*CASS4*	0.88	0.52	1.12	(0.85–1.48)	.09	0.10	0.09

OR, odds ratio; MAF, minor allele frequency; CI, confidence interval.
