# Supplementary material for: Genetic risk factors for the posterior cortical atrophy variant of Alzheimer's disease
Source: Alzheimers Dement. 2016 Aug;12(8):862–71. doi: 10.1016/j.jalz.2016.01.010 (PMC4982482; doi:10.1016/j.jalz.2016.01.010)
Supplement: Supplementary Figures 1 and 2 and Supplementary Tables 1 and 2 [file mmc1.docx]

Supplementary Figure 1

**Multiple Dimensional Scaling Plot**

A plot of the first two dimensions (PC1 and PC2) of a multiple dimensional scaling analysis implemented using PLINK and 100,000 SNPs common to all genotyping platforms in the study. This illustrates that our controls populations are adequately representative of the PCA cases. All outliers have been removed in the plot shown.

Supplementary Figure 2.

**Quantile-quantile plot**

Shows only modest genomic inflation of the association test statistic (λ_GC_=1.06). Four population covariates were used in the regression test implemented in SNPTESTv2.5β4. Observed data are shown as black dots, expected data are shown as a red line, 95% confidence zone is shown in grey.


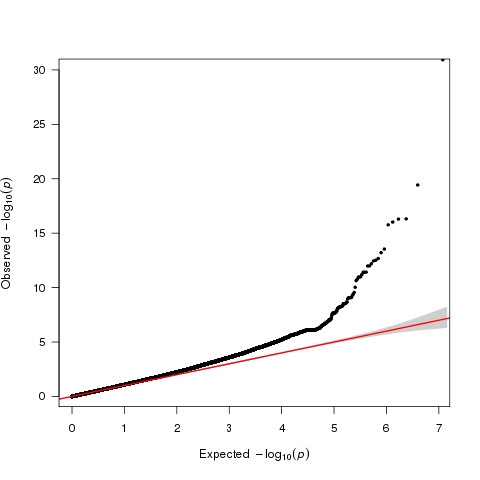


Supplementary Table 1

**Sample Exclusions**

| Sample Cohort | Chip Type | Total Number Genotyped | Number Excluded Call Rate <98% | Number Duplicates Excluded From Re-examination Of Patient Notes | Number Excluded IBS Estimate Pi-Hat >0.1875 | Number Removed as Outliers Upon MDS Visualisation | Total Number Remaining for Analysis |
| --- | --- | --- | --- | --- | --- | --- | --- |
| PCA cases | Illumina 660K | 57 | 1 | 1* | 0 | 2 | 54 |
| PCA cases | Omni Express | 245 | 5 | 1* | 1 | 0 | 239 |
| KORAF4 German controls | Illumina 550K | 840 | 17 | 0 | 6 | 8 | 809 |
| KORAF3 German controls | Illumina 2.5M | 1950 | 3 | 0 | 57 | 8 | 1882 |
| Geisinger US controls | Omni Express | 1264 | 2 | 0 | 69 | 8 | 1185 |
| Framingham US controls | Illumina 5M | 2467 | 16 | 0 | 793 | 7 | 1651 |
| WTCCC2 controls | Illumina 1.2M | 5050 | 26 | 0 | 4 | 0 | 5020 |

*Both PCA cases discovered to be duplicates upon re-examination of further patient data (made available post genotyping) were also excluded for low call rate. Thus the total number of PCA exclusions was 9.

Supplementary Table 2

This table includes the SNPTEST output from the SNPs represented on all arrays (ie the intersection of all array types, not imputed data) with an association P value <10-4. For information metrics (“average maximum posterior call” and “info”) please refer to online literature associated with the SNPTEST software available at https://mathgen.stats.ox.ac.uk/genetics_software/snptest/old/snptest_v2.3.0.html.

HWE=Hardy-Weinberg Equilibrium, MAF=minor allele frequency.
